# Captiva EMR-Lipid柱净化-超高效液相色谱-串联质谱法快速同时测定谷物及其制品中11种麦角生物碱

**DOI:** 10.3724/SP.J.1123.2024.02022

**Published:** 2025-04-08

**Authors:** Bolin LIU, Dan ZHANG, Ziwei ZHAO, Ji’an XIE, Ziyue ZHAN, Qi ZHANG, Weidong LI

**Affiliations:** 1.安徽省疾病预防控制中心,安徽 合肥 230601; 1. Anhui Provincial Center for Disease Control and Prevention, Hefei 230601, China; 2.安徽医科大学,安徽 合肥 230000; 2. Anhui Medical University, Hefei 230000, China

**Keywords:** 超高效液相色谱-串联质谱, 谷物及其制品, 麦角生物碱, 真菌毒素, ultra performance liquid chromatography-tandem mass spectrometry (UPLC-MS/MS), cereals and their products, ergot alkaloids (EAs), mycotoxins

## Abstract

麦角生物碱(EAs)是由麦角菌属(*Claviceps*)产生的一类真菌毒素,当人类摄入被EAs污染的谷物及其制品时,可能会遭受慢性或急性中毒,从而威胁身体健康。鉴于此,本文基于超高效液相色谱-串联质谱(UPLC-MS/MS)建立了谷物及其制品中11种EAs的灵敏、快速测定方法。采用20 mL乙腈-200 mg/L碳酸铵溶液(80∶20, v/v)对谷物及其制品中的11种EAs进行提取,在10000 r/min下离心10 min,用通过式Captiva EMR-Lipid净化柱对上清液进行净化。采用ACQUITY UPLC HSS T3色谱柱(100 mm×3 mm, 1.8 μm)分离,以1 mmol/L乙酸铵溶液和乙腈为流动相进行梯度洗脱。在电喷雾电离(ESI)正离子模式下,采用多反应监测(MRM)模式采集数据,用基质匹配标准曲线进行外标法定量。方法学验证表明,11种EAs在各自的线性范围内具有良好的线性关系,相关系数(*r*^2^)为0.9933~0.9999,检出限为0.006~0.2 μg/kg,定量限为0.02~0.6 μg/kg。以小麦粉、薏苡仁、小麦粉制品和玉米粉为基质样品,在低、中、高3个加标水平下,11种EAs的回收率为80.1%~118%,相对标准偏差(RSD)为0.2%~13.3%。将所建方法用于市售谷物及其制品(240份小麦粉、80份玉米粉、30份大米、30份薏苡仁及146份小麦粉制品)中EAs的含量测定。结果表明,每种样品均有EAs检出,11种EAs的检出率为0.57%~20.3%。该方法具有简单、快速、准确等优势,适用于谷物及其制品中多种EAs的快速同时测定。

## Abstract (en)

Ergot alkaloids (EAs) are mycotoxins produced by *Claviceps* and are present in cereals and their products; their residues pose significant threats to human health through food consumption, resulting in ergotism and sickness. Herein, a sensitive and rapid method for the determination of 11 EAs in cereals and their products using ultra performance liquid chromatography-tandem mass spectrometry (UPLC-MS/MS) were developed. Eleven EAs were extracted with 20 mL acetonitrile-200 mg/L ammonium acetate solution (80∶20, v/v) for 15 min using the vortex shock method followed by 15 min of ultrasonication. The mixture was subsequently centrifuged at 10000 r/min for 10 min, and the supernatant was purified by a Captiva EMR-Lipid column. Target analytes were separated on an ACQUITY UPLC HSS T3 chromatography column (100 mm×3 mm, 1.8 μm) at a column temperature of 40 ℃ and a flow rate of 0.4 mL/min using an injection volume of 5 μL. Gradient elution was performed using 1 mmol/L ammonium acetate solution and acetonitrile as mobile phases. Data were collected in electrospray positive-ion (ESI^+^) and multi-reaction monitoring (MRM) modes, and quantified using matrix-matched standard curves. The 11 EAs exhibited good linearities in their linear ranges, with correlation coefficients (*r*^2^) of 0.9933-0.9999, with limits of detection (LODs) and limits of quantification (LOQs) of 0.002-0.2 and 0.006-0.6 μg/kg, respectively. Recoveries and relative standard deviations (RSDs) of the 11 EAs in matrix samples of wheat flour, coix seed, wheat flour products, and corn flour at low, medium, and high spiked levels were 80.1%-118% and 0.2%-13.3%, respectively. The established method was used to determine EAs in 240 wheat flour, 80 corn flour, 30 rice, and 30 coix seed samples, as well as 146 wheat flour products, with the detection rates of the 11 EAs of 0.57%-20.3%. A maximum total content of EAs of 56.7 μg/kg was recorded for a single sample. The sample pretreatment process used in this method is simple and fast, and the detection method is highly sensitive, with accurate and reliable results obtained. This method is suitable for simultaneously determining various EAs in cereals and their products. The results of this study provide valuable information for future EA risk-assessment studies.

麦角生物碱(ergot alkaloids, EAs)是由麦角菌属(*Claviceps*)产生的一类真菌毒素。麦角菌容易寄生在谷物(如小麦、黑麦、燕麦等)的子房内,取代原本的谷粒或种子形成菌核,这种菌核被称之为麦角,EAs主要来源于麦角。据报道,加拿大、荷兰和比利时种植的谷物中均检出了EAs^[1⇓-3]^;意大利和阿尔及利亚生产的以谷物为主要成分的婴儿食品中,EAs含量超出了欧盟规定的限值(20 μg/kg)^[4⇓-6]^;在斯洛文尼亚,约有17%的谷物被EAs污染^[7]^。Shi等^[8]^报道称,在牲畜生产商送检的冬季大麦和小麦样品中,有91%的大麦样品和84%的小麦样品中至少检出了一种EAs。同样,我国也有关于粮谷中检出EAs的报道^[9]^。由此可见,全球范围内的谷物食品均受到了EAs的污染。

动物试验结果表明,EAs对动物平滑肌细胞具有毒性作用,且随着EAs水平的升高,其对细胞的生长抑制作用也随之增强^[10]^。兔子食用含有EAs的饲料后,其尾部会出现局部缺血坏死的现象^[11]^。EAs能够引发血管收缩,进而影响牛中肠的营养吸收^[12]^。当EAs的摄入量超过2447 μg/kg时,绵羊的生长和代谢会受到影响^[13]^。EAs会导致处于妊娠期的母羊血管收缩,从而影响胎儿的生长和肌肉发育^[14,15]^。当蛋鸡暴露于高水平的EAs时,其产蛋量会显著降低^[16]^。此外,EAs也会对仔猪的生产能力产生不利影响^[17]^。食用被EAs污染的谷物会使人类面临中毒风险^[18]^,可能患痉挛性麦角病等疾病。2021年,联合国粮食与农业组织(FAO)和世界卫生组织(WHO)联合将膳食中EAs的每日可耐受摄入量(TDI)调整为0.4 μg/kg bw;《粮食卫生标准的分析方法》(GB/T 5009.36-2003)^[19]^规定粮食中的麦角不能超过0.1 g/kg;《食品安全国家标准 粮食》(GB 2715-2016)^[20]^规定大米、玉米和豆类中不得检出麦角,小麦、燕麦和大麦等谷物中的麦角含量的质量分数应小于0.01%。上述国家标准中的检测方法均是通过形态特征观察和组织切片技术对麦角进行鉴定,然后采用比色法对EAs进行定性分析。然而目前尚未有关于谷物及其制品中EAs的最大残留限量规定及定量分析的标准检测方法。

目前,液相色谱-串联质谱(LC-MS/MS)已被广泛应用于谷物及其制品中EAs的含量检测^[3,21⇓⇓⇓⇓-26]^,如《出口粮谷中6种麦角碱的测定 液相色谱-质谱/质谱法》(SN/T 4524-2016)^[27]^和《牧草中15种生物碱的测定 液相色谱-串联质谱法》(NY/T 2769-2015)^[28]^采用LC-MS/MS分别测定了粮谷中的6种EAs和牧草中的8种EAs。Cherewyk等^[21]^将氘代麦角二乙酰胺(LSD-D_3_)作为同位素内标,建立了高效液相色谱-串联质谱(HPLC-MS/MS)测定小麦中多种EAs含量的方法,该方法的仪器检出限(LOD)为0.00893~0.225 μg/kg,在小麦基质中,12种EAs的基质效应为101%~113%。Huybrechts等^[3]^基于HPLC-MS/MS,建立了一种婴儿谷物食品中EAs总含量的分析测定方法,该方法能够实现6种EAs的定量测定,所获得的LOD为0.5 ng/g。上述方法大多使用多功能MycoSep 150固相萃取柱或QuEChERS前处理技术。多功能MycoSep 150固相萃取柱是EAs的专用萃取柱,其选择性强,但该柱可萃取的EAs种类较少且存在处理流程繁琐、耗时长等问题^[9]^; QuEChERS法虽简便、快速,但不同类型的吸附剂在吸附杂质的同时也会吸附待测物,极大地限制了可检测的EAs种类。因此,开发一种快速、灵敏、准确的谷物及其制品中多种EAs分析测定方法至关重要。

基于上述问题,本研究将Captiva EMR-Lipid柱净化与超高效液相色谱-串联质谱(UPLC-MS/MS)结合,建立了同时测定谷物及其制品中11种EAs(包括7种*S*构型和4种*R*构型)的方法。该方法具有较高的灵敏度和准确度,样品前处理过程经济、简便,为谷物及其制品中EAs的风险评估和残留限值的制定提供了技术支持。

## 1 实验部分

### 1.1 仪器、试剂与材料

ACQUITY^TM^ UPLC超高效液相色谱-XevoTQ-S串联质谱仪(美国Waters公司); Heidolph多点振荡器(德国Heidolph公司); Legend Mach1.6R高速冷冻离心机(美国Thermo Fisher公司); KN295 Knifetec^TM^刀式样品磨(丹麦Foss公司); BJ-800A高速粉碎机(中国邦杰公司)。

11 种EAs单标储备液:麦角考宁(100 μg/mL, *R*型)、麦角异柯宁碱(25 μg/mL, *S*型)、麦角辛(100 μg/mL, *R*型)、麦角辛宁(25 μg/mL, *S*型)、麦角异卡里碱(100 μg/mL, *S*型)、麦角隐亭(100 μg/mL, *R*型)、麦角克碱(100 μg/mL, *R*型)、麦角异克碱(25 μg/mL, *S*型)、双氢麦角汀(100 μg/mL, *R*型)、麦角胺(100 μg/mL, *R*型)和马来酸麦角新碱(100 μg/mL, *R*型)购自奥地利Romer Labs公司。甲醇、乙腈、甲酸和乙酸(HPLC级,德国Merck公司);乙酸铵、甲酸铵、碳酸铵(HPLC级,美国Sigma-Aldrich公司); *N*-丙基乙二胺(PSA)吸附剂、C_18_吸附剂(50 μm)、Bond Elut EMR-Lipid净化试剂包和Captiva EMR-Lipid净化柱(3 mL, 300 mg)(美国Agilent公司); Oasis PRiME HLB固相萃取柱(3 mL, 150 mg)(美国Waters公司);实验用水均为Milli-Q纯水机(美国Millipore公司)制备的超纯水(18.2 MΩ·cm)。

谷物及其制品(包括小麦粉、大米、薏苡仁、玉米糁、玉米粉、馒头、饺子皮、包子、面条)采集于本省超市和农贸市场。

### 1.2 实验条件

#### 1.2.1 标准溶液的配制

准确移取适量11种EAs单标储备液于棕色容量瓶中,用乙腈定容,配制成混合标准使用液,其中麦角异克碱、麦角隐亭、麦角辛宁和麦角异柯宁碱的质量浓度为100 μg/L,双氢麦角汀、麦角克碱、麦角考宁、麦角异卡里碱和麦角胺的质量浓度为200 μg/L,麦角辛和马来酸麦角新碱的质量浓度为1000 μg/L。用乙腈-200 mg/L碳酸铵溶液(80∶20, v/v)稀释上述混合标准使用液,配制成系列质量浓度的混合标准工作液,其中麦角异克碱、麦角隐亭、麦角辛宁和麦角异柯宁碱的质量浓度均为5.0 μg/L,双氢麦角汀、麦角克碱、麦角考宁、麦角异卡里碱和麦角胺的质量浓度均为10.0 μg/L,麦角辛和马来酸麦角新碱的质量浓度均为100 μg/L。

#### 1.2.2 样品采集与制备

在本省超市和农贸市场采集谷物及小麦粉制品,每份样品均不少于500 g;其中大米和薏苡仁等颗粒样品均经刀式样品磨打碎,混匀;小麦粉制品样品(如馒头、饺子皮、包子、面条)预先在-20 ℃冰箱中冷冻为硬块,随后采用高速粉碎机打碎,混匀。采用四分法将粉碎后的样品分成两份(用于分析和复测),分别装入食品级保鲜盒中,密封后标记好编号,并置于-20 ℃冰箱中避光保存。

#### 1.2.3 样品前处理

分别准确称取5.0 g(精确到0.001 g)上述试样于50 mL离心管中,加入20 mL乙腈-200 mg/L碳酸铵溶液(80∶20, v/v),涡旋振荡15 min,再超声提取15 min,在4 ℃下以10000 r/min离心10 min,将上清液转移至新的50 mL离心管中;用乙腈-200 mg/L碳酸铵溶液(80∶20, v/v)将上述溶液稀释至20 mL,混匀后移取3 mL上清液过Captiva EMR-Lipid净化柱,以1滴/s的速度收集滤液,滤液经再生纤维膜(RC)过滤后,供UPLC-MS/MS分析。

### 1.3 分析条件

#### 1.3.1 色谱条件

色谱柱:ACQUITY UPLC HSS T3柱(100 mm×3 mm, 1.8 μm);流动相A为1 mmol/L乙酸铵溶液,流动相B为乙腈;流速:0.4 mL/min;进样体积:5 μL;柱温:40 ℃;样品室温度:10 ℃。梯度洗脱程序:0~3.0 min, 40%A~75%A; 3.0~4.0 min, 75%A~10%A; 4.0~5.0 min, 10%A; 5.0~6.0 min, 10%A~40%A; 6.0~8.0 min, 40%A。

#### 1.3.2 质谱条件

离子源:电喷雾电离(ESI)源,正离子扫描模式;毛细管电压:1.5 kV;离子源温度:150 ℃;脱溶剂气温度:500 ℃;脱溶剂气流量:1000 L/h;碰撞气流量:0.15 mL/min;检测模式:多反应监测(MRM)。其他质谱参数见表1。

**表1:** 11种EAs的保留时间和质谱参数

Compound	Retention time/ min	Parent ion (*m/z*)	Product ions (*m/z*)	CE/eV	CVs/V
Ergometrine maleate (马来酸麦角新碱)	1.01	326.3	223.2^*^, 208.0	38	24, 28
Ergosine (麦角辛)	1.89	548.4	223.2^*^, 268.2	34	32, 24
Ergotamine (麦角胺)	2.20	582.4	223.2^*^, 208.1	36	30, 44
Ergocornine (麦角考宁)	4.59	562.4	268.2^*^, 208.1	40	26, 44
Dihydroergocristine (双氢麦角汀)	4.70	612.4	270.2^*^, 168.1	40	30, 60
Ergocryptine (麦角隐亭)	4.79	576.4	223.2^*^, 268.2	40	35, 26
Ergocristine (麦角克碱)	4.83	610.4	223.2^*^, 208.1	40	36, 48
Ergosinine (麦角辛宁)	4.97	548.4	223.2^*^, 277.2	40	32, 26
Ergocorninine (麦角异柯宁碱)	5.17	562.4	277.2^*^, 223.2	38	26, 34
Ergocryptinine (麦角异卡里碱)	5.30	576.4	223.2^*^, 305.2	40	36, 28
Ergocristinine (麦角异克碱)	5.34	610.4	305.2^*^, 325.2	40	28, 28

^*^ Quantitative ion; CE: collision energy; CV: cone voltage.

## 2 结果与讨论

### 2.1 UPLC-MS/MS条件的优化

#### 2.1.1 质谱条件的优化

分别配制系列质量浓度的11种EAs混合标准工作液,以乙腈和1 mmol/L乙酸铵溶液为流动相,采用针泵进样方式进样,在ESI源下获得高丰度的分子离子。结果表明,11种EAs的[M+H]^+^响应值明显高于[M+NH_4_]^+^和[M+Na]^+^,因此,确定分子离子峰[M+H]^+^为母离子;进一步进行二级子离子的扫描,选择响应值最高的两个子离子分别作为定量离子和定性离子;利用IntelliStart功能进行质谱参数的优化,获得了11种待测物的子离子、锥孔电压和碰撞能量,详见表1。

#### 2.1.2 色谱柱的优化

实验分别比较了ACQUITY UPLC HSS T3(100 mm×3 mm, 1.8 μm)、CORTECS UPLC C_18_(100 mm×2.1 mm, 1.6 μm)和ACQUITY UPLC BEH C_18_(100 mm×2.1 mm, 1.7 μm)3种色谱柱对待测物分离效果及响应值的影响。结果表明,除麦角辛和马来酸麦角新碱外,其他9种EAs在ACQUITY HSS T3色谱柱上的响应值均高于CORTECS UPLC C_18_柱和ACQUITY UPLC BEH C_18_柱,甚至高出两倍以上。ACQUITY HSS T3色谱柱的填料颗粒度较大,柱效较高,能够使更多分析物进入孔隙,并且其对极性化合物具有更好的保留效果。因此,实验最终选择ACQUITY UPLC HSS T3柱(100 mm×3 mm, 1.8 μm)作为分析柱。

#### 2.1.3 流动相的优化

实验考察了不同流动相体系(水相:0.1%甲酸水溶液、1 mmol/L乙酸铵溶液、1 mmol/L甲酸铵溶液、1 mmol/L碳酸铵溶液,有机相均为乙腈)对11种EAs色谱分离度和检测灵敏度的影响。实验结果表明,当流动相水相为0.1%甲酸水溶液时,麦角考宁与麦角异柯宁碱等同分异构体化合物出现了双峰且分离度较差;当流动相水相为1 mmol/L乙酸铵溶液时,11种EAs的响应值明显高于1 mmol/L甲酸铵溶液;以1 mmol/L碳酸铵溶液为流动相水相时,11种EAs的响应值明显高于1 mmol/L乙酸铵溶液。虽然11种EAs在1 mmol/L碳酸铵溶液中具有更高的灵敏度,但马来酸麦角新碱存在色谱峰展宽和拖尾的现象,而在1 mmol/L乙酸铵溶液条件下,11种待测物的色谱峰形尖锐且对称。因此,实验最终选择1 mmol/L乙酸铵溶液为流动相水相。此外,实验考察了不同浓度(0.5、1、2、5、10 mmol/L)的乙酸铵溶液对11种EAs检测灵敏度的影响。结果发现,当乙酸铵溶液浓度为0.5 mmol/L时,马来酸麦角新碱在色谱柱上无保留,保留时间仅为0.6 min;当乙酸铵溶液浓度增加至1 mmol/L时,11种EAs的响应值均有提高,且马来酸麦角新碱的保留时间增加至1.01 min;当继续增大乙酸铵溶液的浓度分别至2、5、10 mmol/L时,11种EAs的响应值逐渐下降,且在乙酸铵溶液浓度为10 mmol/L时降至最低。因此,确定乙酸铵溶液的浓度为1 mmol/L。最后,实验分别比较了甲醇和乙腈对11种EAs检测灵敏度的影响,结果表明,除麦角辛外,其他10种EAs在甲醇流动相下的响应值均低于乙腈。综上,确定本实验的最优流动相体系为1 mmol/L乙酸铵溶液和乙腈。在最佳实验条件下,11种EAs的MRM色谱图如图1所示。

**图1:**
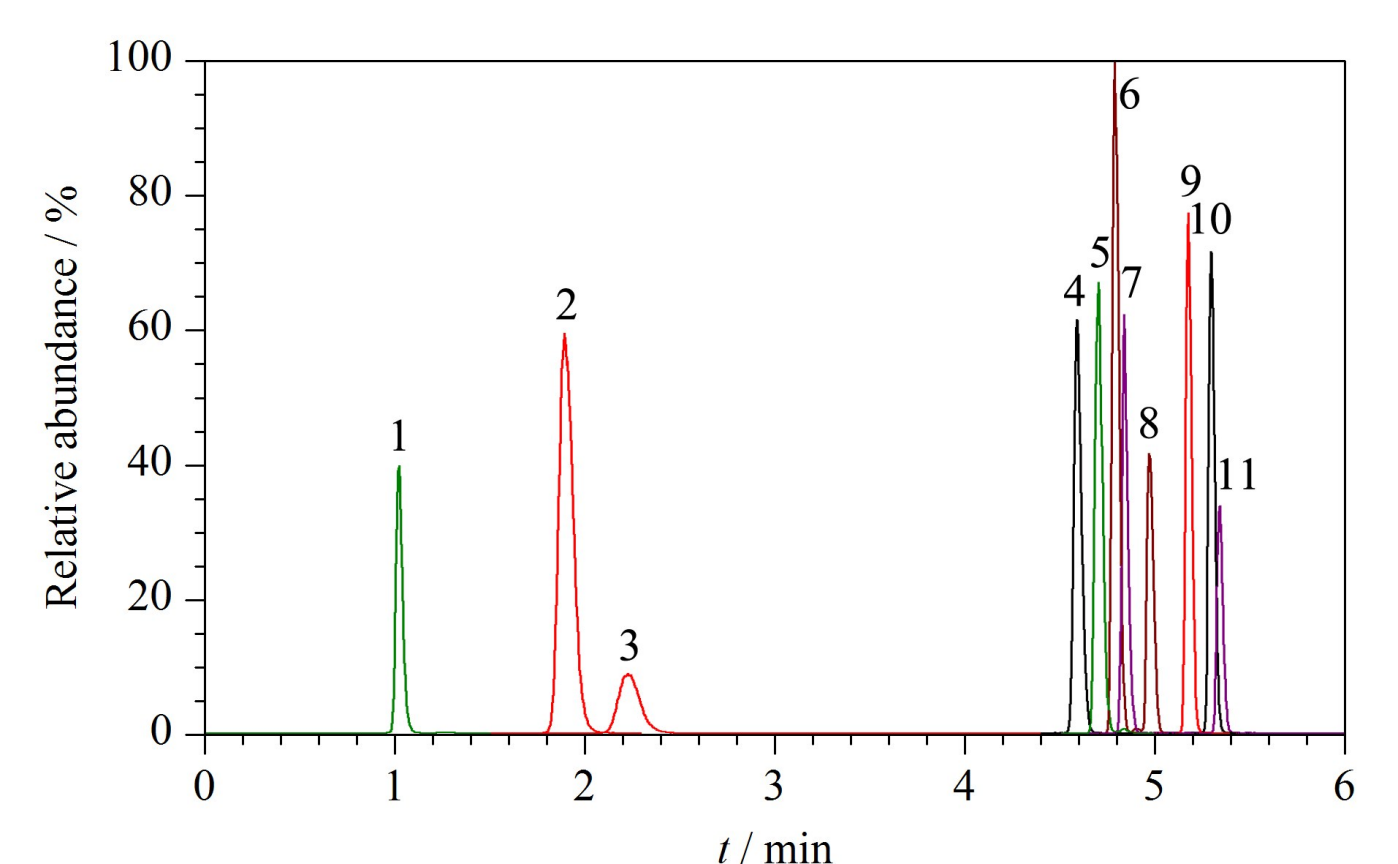
11种EAs混合标准工作液的MRM色谱图

### 2.2 样品前处理条件的优化

#### 2.2.1 提取溶剂的选择

《牧草中15种生物碱的测定 液相色谱-串联质谱法》(NY/T 2769-2015)^[28]^中采用乙腈-200 mg/L碳酸铵溶液(50∶50, v/v)提取牧草中的15种生物碱;汪薇等^[9]^采用乙腈-水(84∶16, v/v)提取粮谷中的9种麦角碱;Huybrechts等^[3]^和Poapolathep等^[22]^采用乙腈-200 mg/L碳酸铵溶液(84∶16, v/v)分别提取婴儿食品和饲料中的EAs。研究表明,麦角胺、麦角考宁、麦角隐亭、麦角克碱等化合物在甲醇中不稳定,在乙腈和乙腈-碳酸铵溶液中较稳定,且不易发生异构化^[9,29]^。结合11种EAs的化学性质及常见真菌毒素的提取溶剂,实验以空白基质加标后的玉米粉基质样品为例(其中麦角异克碱、麦角隐亭、麦角辛宁和麦角异柯宁碱的加标含量为0.4 μg/kg,双氢麦角汀、麦角克碱、麦角考宁、麦角异卡里碱和麦角胺的加标含量为0.8 μg/kg,麦角辛和马来酸麦角新碱的加标含量为4.0 μg/kg),分别考察了乙腈-水(80∶20, v/v)、乙腈-乙酸铵溶液(80∶20, v/v)、乙腈-5%氨水溶液(80∶20, v/v)、乙腈-200 mg/L碳酸铵溶液(80∶20, v/v)和乙腈-400 mg/L碳酸铵溶液(80∶20, v/v)对11种EAs提取效果的影响。实验结果表明,部分EAs在乙腈-水(80∶20, v/v)、乙腈-乙酸铵溶液(80∶20, v/v)、乙腈-5%氨水溶液(80∶20, v/v)中均存在回收率较低的现象。当使用乙腈-5%氨水溶液进行提取时,玉米粉样品提取液的颜色偏黄,可能是因为在碱性条件下玉米粉中的色素被共提取,不利于后续的净化处理。因此,实验重点比较了乙腈-200 mg/L碳酸铵溶液(80∶20, v/v)和乙腈-400 mg/L碳酸铵溶液(80∶20, v/v)对11种EAs的提取效果。结果表明,11种EAs在两种提取溶剂中的回收率分别为77.0%~118%和71.0%~117%,几乎不受碳酸铵溶液浓度的影响。结合绿色化学的理念,本文选择乙腈-200 mg/L碳酸铵溶液进行后续优化实验。

进一步考察了乙腈与200 mg/L碳酸铵溶液的体积比(80∶20、65∶35、50∶50)对11种EAs提取效果的影响。实验结果表明,80∶20、65∶35和50∶50的乙腈-200 mg/L碳酸铵溶液对11种EAs的平均回收率分别为80.3%、55.8%和50.9%,相对标准偏差(RSD,*n*=3)分别为3.55%、4.28%和4.50%。结果表明,使用乙腈-200 mg/L碳酸铵溶液(80∶20, v/v)进行提取时,EAs能更容易地从样品基质中提取出来,因此最终确定提取溶剂为乙腈-200 mg/L碳酸铵溶液(80∶20, v/v)。

#### 2.2.2 净化方式的选择

为了降低谷物及其制品中11种EAs的基质效应、提高方法的准确性,需对样品提取液进行净化处理。本实验以空白基质加标后的玉米粉为基质样品(其中11种EAs的加标含量同2.2.1节),分别考察了C_18_吸附剂、PSA吸附剂、Oasis PRiME HLB固相萃取柱、Bond Elut EMR-Lipid净化试剂包和Captiva EMR-Lipid净化柱等5种净化方式对11种EAs回收率的影响。其中,C_18_吸附剂、PSA吸附剂和Oasis PRiME HLB固相萃取柱按照文献[9]报道方法进行净化实验,Bond Elut EMR-Lipid净化试剂包参照说明书进行净化实验。结果如图2所示,当采用C_18_吸附剂和PSA吸附剂对样品提取液进行净化时,11种EAs的回收率分别为56.7%~132%和48.3%~101%, RSD分别为1.4%~4.5%和3.2%~8.2%,其中麦角辛、麦角考宁、麦角异柯宁碱、麦角异卡里碱和麦角异克碱的回收率偏低,而马来酸麦角碱的回收率偏高,净化效果均不理想。当采用Oasis PRiME HLB固相萃取柱和Bond Elut EMR-Lipid净化试剂包净化时,11种EAs的回收率普遍偏低,分别为17.2%~73.6%和16.7%~54.4%。其中,Bond Elut EMR-Lipid净化试剂包中的脱水盐包在净化过程中会迅速吸水并释放热量,导致样品提取液的温度升高,粉状样品发生结块,进而将待测物包裹在内,这会导致后续的反萃取过程无法完全进行;同时,高温环境也会导致EAs的不稳定性增加,从而造成EAs的大量损失。样品提取液经Captiva EMR-Lipid净化柱净化后,11种EAs的回收率为83.3%~108%, RSD为1.3%~13.2%。因此,最终选择Captiva EMR-Lipid净化柱用于后续净化实验。

**图2:**
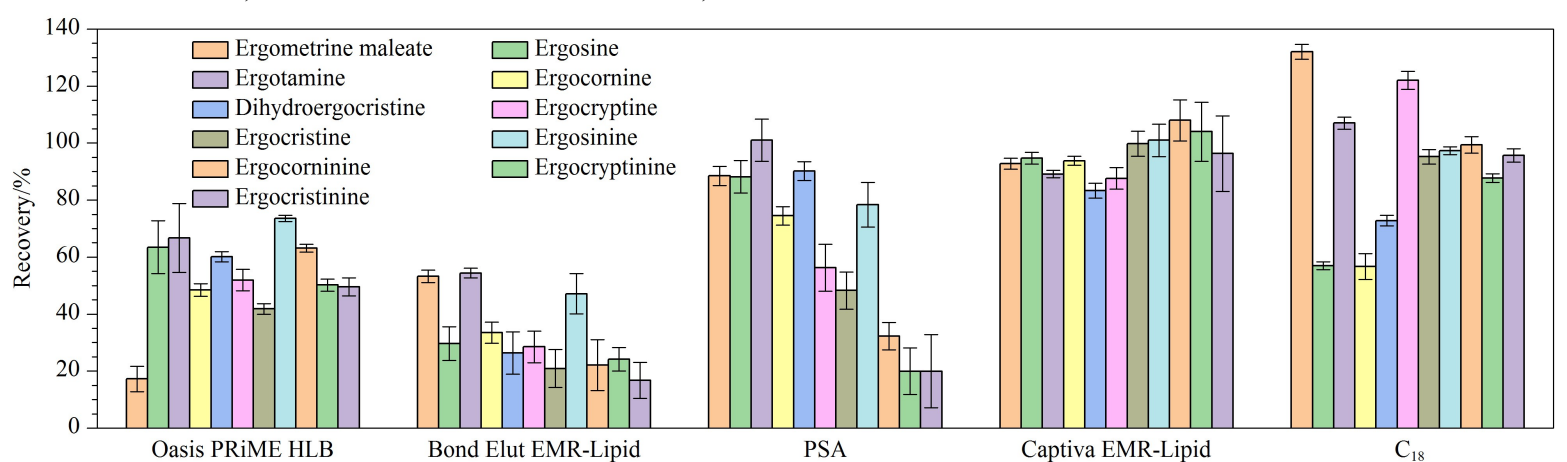
不同净化方式对11种EAs回收率的影响(*n*=3)

### 2.3 基质效应的考察

按照1.2.3节方法分别对4种空白基质样品(小麦粉、薏苡仁、小麦粉制品和玉米粉)进行前处理,获得相应的空白基质提取液。用上述空白基质提取液配制系列质量浓度的基质匹配混合标准溶液,并上机分析。同时,用乙腈-200 mg/L碳酸铵溶液(80∶20, v/v)配制相同质量浓度的溶剂混合标准溶液,并上机分析。采用Besil等^[30]^报道的基质效应(ME)评价方法(ME=(基质匹配标准曲线的斜率/溶剂标准曲线的斜率-1)×100%),对11种EAs的基质效应进行评价。当ME为正值时,表现为基质增强效应;当ME为负值时,表现为基质抑制效应;其中|ME|>50%为强基质效应,20%<|ME|≤50%为中等基质效应,|ME|≤20%为弱基质效应^[30]^。结果如图3所示,在4种样品基质中,多种EAs存在较强的基质效应,|ME|为0.49%~596%。为了降低样品基质效应所带来的干扰,本实验采用基质匹配混合标准溶液进行后续实验。

**图3:**
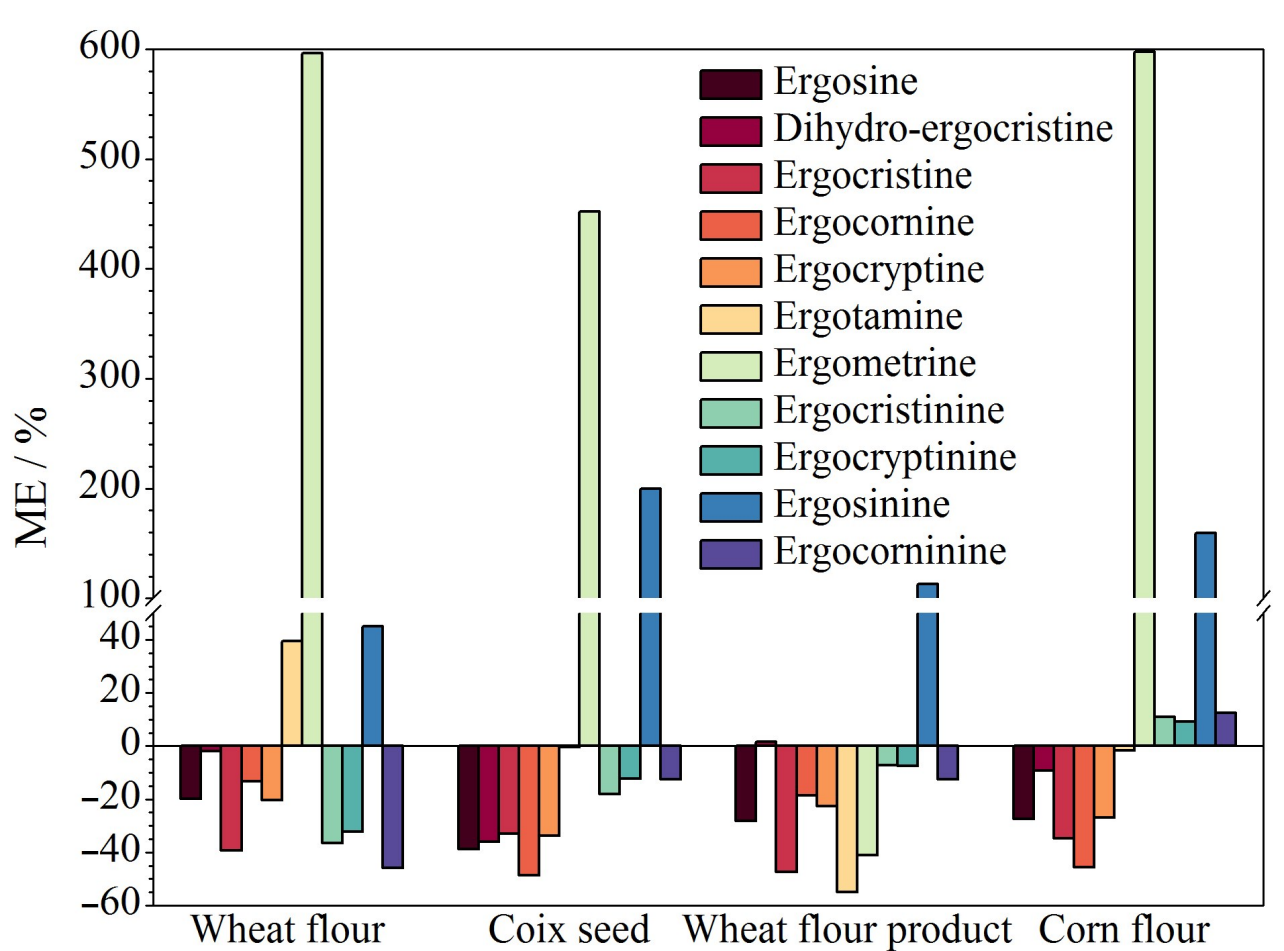
4种谷物及其制品中11种EAs的基质效应

### 2.4 方法学验证

#### 2.4.1 线性范围、检出限和定量限

分别用4种空白基质样品(小麦粉、薏苡仁、小麦粉制品和玉米粉)提取液对11种EAs的混合标准工作液进行逐级稀释,配制成系列质量浓度的基质匹配混合标准溶液,并按照1.3节实验条件进行测定。以待测物的峰面积为纵坐标(*y*),质量浓度为横坐标(*x*, μg/L),绘制基质匹配标准曲线。结果表明,4种基质样品中11种EAs在各自的范围内具有良好的线性关系,线性相关系数(*r*^2^)为0.9933~0.9999。在小麦粉、薏苡仁、小麦粉制品和玉米粉4种空白基质样品中分别添加基质匹配标准曲线中最低质量浓度所对应的混合标准工作液,按照1.2.3节方法进行样品前处理后上机测定。分别以3倍信噪比(*S/N*)和10倍*S/N*对应的含量计算检出限(LOD)和定量限(LOQ), 4种基质样品中11种EAs的LOD和LOQ分别为0.006~0.2 μg/kg和0.02~0.6 μg/kg,小麦粉基质样品的相关数据结果见表2,其他数据见附表1(https://www.chrom-China.com)。以空白小麦粉基质样品为例,加标前后的MRM色谱图见附图1。目前,我国《食品安全国家标准 食品中真菌毒素限量》(GB 2761-2017)^[31]^中尚未规定谷物及其制品中EAs含量及其总量的限量值,仅在《粮食卫生标准的分析方法》(GB/T 5009.36-2003)^[19]^中规定了粮食中麦角的添加量不得超过0.1 g/kg。欧盟委员会发布的食品中EAs最高残留限量的法规((EU)2023/195)^[4]^规定婴幼儿谷类加工食品中EAs的最高残留限量为20 μg/kg,投放到市场的大麦、小麦、斯佩耳特小麦和燕麦研磨产品中EAs的最高残留限量为150 μg/kg。参照上述国家标准与相关法规,本方法中11种EAs的LOQ均满足定量分析的要求。

**表2:** 小麦粉基质样品中11种EAs的线性范围、线性方程、相关系数、检出限和定量限

Analyte	Linear range/(μg/L)	Linear equation	*r*^2^	LOD/(μg/kg)	LOQ/(μg/kg)
Ergometrine maleate	0.01-50	*y*=2.80×10^5^*x*+4.18×10^2^	0.9969	0.06	0.2
Ergosine	0.01-50	*y*=9.24×10^4^*x*+2.12×10^2^	0.9988	0.06	0.2
Ergotamine	0.002-10	*y*=2.02×10^5^*x*-7.54×10^1^	0.9991	0.013	0.04
Ergocornine	0.002-10	*y*=2.99×10^5^*x*+1.64×10^2^	0.9998	0.013	0.04
Dihydroergocristine	0.002-10	*y*=3.82×10^5^*x*+3.76×10^2^	0.9994	0.013	0.04
Ergocryptine	0.002-10	*y*=4.06×10^5^*x*+4.78×10^2^	0.9994	0.013	0.04
Ergocristine	0.002-10	*y*=1.63×10^5^*x*+4.98×10^2^	0.9977	0.013	0.04
Ergosinine	0.001-5	*y*=6.89×10^5^*x*+2.43×10^2^	0.9998	0.006	0.02
Ergocorninine	0.001-5	*y*=3.88×10^5^*x*+4.41×10^2^	0.9993	0.006	0.02
Ergocryptinine	0.001-5	*y*=4.78×10^5^*x*+5.23×10^2^	0.9995	0.006	0.02
Ergocristinine	0.001-5	*y*=2.10×10^5^*x*+6.04×10^2^	0.9996	0.006	0.02

*y*: peak area; *x*: mass concentration, μg/L.

#### 2.4.2 准确度和精密度

为了考察本方法的准确度和精密度,参考(EU)2023/195^[4]^和GB/T 27404-2008^[32]^,在低、中、高3个加标水平下,对空白小麦粉、薏苡仁、小麦粉制品和玉米粉基质样品进行加标回收试验,每个样品平行测定6次,计算回收率和RSD。结果如表3所示,4种基质样品中11种EAs的回收率为80.1%~118%, RSD为0.2%~13.3%,表明该方法的准确度和精密度良好。

**表3:** 4种不同基质样品中11种EAs的回收率和相对标准偏差(*n*=6)

Analyte	Spiked level/ (μg/kg)		Recoveries/%										RSDs/%							
			Wheat flour		Coix seed		Wheat flour product		Corn flour				Wheat flour		Coix seed		Wheat flour product		Corn flour	
Ergometrine maleate		0.2		91.7		93.9		106		103				2.2		0.7		4.1		7.0
		40		83.5		88.6		90.2		102				1.1		1.3		1.3		1.2
		200		96.3		93.3		91.8		97.5				0.2		1.6		3.2		0.7
Ergosine		0.2		90.7		90.8		81.7		93.0				1.2		0.8		5.8		1.5
		40		98.1		101		95.9		96.8				1.2		1.5		1.4		0.5
		200		94.3		98.8		105		90.7				1.5		1.9		0.9		1.5
Ergotamine		0.04		90.0		95.8		87.3		100				6.2		4.2		4.9		2.0
		8		92.8		99.7		83.7		97.4				0.2		0.4		3.6		0.8
		40		92.3		98.9		102		96.0				1.3		0.3		12.2		0.7
Ergocornine		0.04		89.7		94.7		87.0		92.0				5.5		9.9		1.1		4.7
		8		102		98.5		118		96.7				1.6		1.8		1.6		1.0
		40		101		101		115		92.7				0.7		0.8		0.8		0.5
Dihydroergocristine		0.04		98.7		80.1		102		92.9				1.4		7.0		2.1		3.4
		8		89.1		99.1		103		82.9				0.4		1.0		1.6		1.1
		40		89.4		80.8		97.4		100				0.5		5.4		1.7		1.4
Ergocryptine		0.04		87.3		90.7		86.7		80.3				4.8		5.7		1.3		3.8
		8		95.8		84.8		111		90.5				1.2		2.0		2.2		1.1
		40		91.7		82.9		103		83.1				1.0		1.9		0.4		1.7
Ergocristine		0.04		88.0		82.3		100		96.4				10.8		3.1		2.6		5.6
		8		99.9		93.5		112		95.1				0.7		1.7		3.7		0.9
		40		97.6		88.9		111		90.2				1.4		0.7		1.5		2.9
Ergosinine		0.02		86.7		102		107		85.0				12.5		1.6		2.7		5.1
		4		83.3		95.1		104		89.9				0.9		6.9		1.0		2.9
		20		82.0		87.8		99.4		82.7				2.2		4.8		6.9		1.7
Ergocorninine		0.02		110		103		107		82.9				6.6		2.2		1.9		6.7
		4		115		82.0		111		89.1				1.5		10.4		4.2		2.4
		20		114		83.4		105		94.9				3.2		7.2		13.1		1.6
Ergocryptinine		0.02		82.7		94.7		81.7		85.9				13.3		6.5		4.7		6.5
		4		96.1		80.7		97.5		89.1				1.2		7.6		1.4		2.9
		20		100		87.8		112		92.0				7.9		10.2		3.5		0.9
Ergocristinine		0.02		85.3		85.0		85.0		87.3				8.9		5.9		11.8		9.5
		4		102		84.8		97.3		91.6				1.5		3.4		1.6		3.6
		20		103		80.3		108		91.9				2.7		11.8		6.0		0.7

### 2.5 实际样品的测定

为了验证本方法的实用性,采用本方法对526份市售谷物及其制品样品中的11种EAs进行测定。实验结果表明,每种样品均有EAs检出,11种EAs的检出率为0.57%~20.3%;麦角异克碱、麦角克碱、麦角异卡里碱、麦角异柯宁碱和麦角隐亭的检出率最高,分别为20.3%、20.0%、16.4%、16.0%和10.3%,检出含量为0.004~22.2 μg/kg;单份样品中检出的EAs总含量最高达56.7 μg/kg。此外,有54.8%的样本检出单一的EAs, 20.3%的样本检出2种EAs, 10.1%的样本检出3种EAs,表明谷物及其制品中存在一定程度的EAs共污染现象。从样品种类来看,小麦粉受到麦角异克碱、麦角克碱、麦角异卡里碱和麦角异柯宁碱4种EAs的污染,小麦粉制品(馒头、饺子皮、包子、面条)受到麦角克碱与麦角胺的污染,大米和薏苡仁样品主要受到麦角异克碱的污染,玉米粉样品主要受到麦角隐亭、麦角异卡里碱和麦角辛宁的污染。据文献[21]报道,加拿大生产的小麦样品中,EAs的最大污染水平为942 μg/kg,相比之下,本文测试的526份样品中EAs的检出含量较低,这可能与当地的气候条件有关。

## 3 结论

本文基于UPLC-MS/MS技术和Captiva EMR-Lipid净化柱,建立了同时测定谷物及其制品中11种EAs的方法。该方法的样品前处理过程简便、快捷,准确度与精密度高。将该方法用于526份市售谷物及其制品中EAs的含量测定,结果发现部分样品存在一定程度的EAs共污染现象。该方法为EAs的风险评估提供了数据基础,同时也为食品安全风险监测提供了技术支持。
